# MSC-Derived Exosome Promotes M2 Polarization and Enhances Cutaneous Wound Healing

**DOI:** 10.1155/2019/7132708

**Published:** 2019-09-09

**Authors:** Xiaoning He, Zhiwei Dong, Yina Cao, Han Wang, Shiyu Liu, Li Liao, Yan Jin, Lin Yuan, Bei Li

**Affiliations:** ^1^State Key Laboratory of Military Stomatology & National Clinical Research Center for Oral Diseases & Shaanxi International Joint Research Center for Oral Diseases, Center for Tissue Engineering, School of Stomatology, Fourth Military Medical University, Xi'an, Shaanxi 710032, China; ^2^Xi'an Institute of Tissue Engineering and Regenerative Medicine, Xi'an, Shaanxi 710032, China; ^3^State Key Laboratory of Military Stomatology & National Clinical Research Center for Oral Diseases & Shaanxi Engineering Research Center for Dental Materials and Advanced Manufacture, Department of Oral Implants, School of Stomatology, The Fourth Military Medical University, Xi'an, Shaanxi 710032, China; ^4^Department of Stomatology, The First Affiliated Hospital of Guangzhou Medical University, Guangzhou, Guangdong 510140, China

## Abstract

Mesenchymal stem cell transplantation (MSCT) promotes cutaneous wound healing. Numerous studies have shown that the therapeutic effects of MSCT appear to be mediated by paracrine signaling. However, the cell-cell interaction during MSCT between MSCs and macrophages in the region of cutaneous wound healing is still unknown. In this study, early depletion of macrophages delayed the wound repair with MSC injection, which suggested that MSC-mediated wound healing required macrophages. Moreover, we demonstrated that systemically infused bone marrow MSCs (BMMSCs) and jaw bone marrow MSCs (JMMSCs) could translocate to the wound site, promote macrophages toward M2 polarization, and enhance wound healing. *In vitro* coculture of MSCs with macrophages enhanced their M2 polarization. Mechanistically, we found that exosomes derived from MSCs induced macrophage polarization and depletion of exosomes of MSCs reduced the M2 phenotype of macrophages. Infusing MSCs without exosomes led to lower number of M2 macrophages at the wound site along with delayed wound repair. We further showed that the miR-223, derived from exosomes of MSCs, regulated macrophage polarization by targeting pknox1. These findings provided the evidence that MSCT elicits M2 polarization of macrophages and may accelerate wound healing by transferring exosome-derived microRNA.

## 1. Introduction

Mesenchymal stem cells (MSCs) are an enticing potential therapeutic agent for a variety of inflammatory reactions, including those that occur during wound healing. Mesenchymal stem cell transplantation (MSCT) is currently being used as a cellular therapy to promote cutaneous wound healing [1–3]. During cutaneous wound healing, most of the therapeutic benefits of MSCT appear to be derived from the release of paracrine factors, which stimulate differentiation and angiogenesis [1]. The cell-cell interaction also plays an important role in promoting wound healing during MSCT [3, 4]. However, the interaction of MSCs and other cells which functionally affect cutaneous wound healing remains to be elucidated.

Although widely recognized as the contributors of the early inflammatory response, monocytes and macrophages also contribute to angiogenesis, wound contraction, and tissue remodeling, which are required in the wound-healing process [5, 6]. In response to activation signals, macrophages are polarized toward an M1 phenotype (proinflammatory) or an M2 phenotype (anti-inflammatory). Accumulating evidence shows that M2 macrophages can express mediators that are essential in the resolution of inflammation and tissue remodeling and, thus, promote wound healing [7, 8]. Several studies have demonstrated that MSCs can modify macrophages from the M1 to the M2 phenotype *in vitro* and *in vivo* [4, 9]. However, the underlying mechanism of the MSC-guided transition of macrophages from the M1 to the M2 phenotype during wound healing is still unknown.

Recently, MSCs have been found to secrete significant amounts of small vesicles (40-100 nm), known as exosomes following fusion of multivesicular endosomal membranes with the cell surface [10, 11]. Exosomes are emerging as a new mechanism for cell-to-cell communication and played an important role in wound repair [12, 13]. They carry a variety of proteins, mRNAs, and microRNAs, all of which may functionally modify recipient cells that interact with exosomes. We hypothesized that exosomes derived from bone marrow-derived mesenchymal stem cells (BMMSCs) mediate the polarization of the M2 macrophage during wound repair.

## 2. Materials and Methods

### 2.1. Animals and Ethical issues

Adult C57BL/6J mice (female, 6 to 8 weeks old) were obtained from the Laboratory Animal Research Center of the Fourth Military Medical University. Animals were maintained under good ventilation and a 12 h light/dark cycle and kept feeding and drinking *ad libitum* before being sacrificed. Mice were anesthetized with 1% pentobarbital sodium (200 mg/kg) via intraperitoneal administration and kept at an anesthetized state during surgery. Animals were euthanized by exsanguinations after receiving intravenous injections of MSCs or exosomes.

All animal procedures were performed according to the guidelines of the Animal Care Committee of Fourth Military Medical University (IRB-REV-2015005), and all experimental protocols were performed with the approval of the Fourth Military Medical University.

### 2.2. Cell Cultures

Human jaw bone marrow-derived mesenchymal stem cells (JMMSCs) and BMMSCs were isolated and identified as previously described [14]. Briefly, JMMSCs and BMMSCs were collected from bone marrow aspirates of the jaw bone and iliac crest, respectively. Bone marrow aspirates were collected, and the cells were plated into 6-well culture dishes (Costar®; Corning Inc., Corning, NY, USA) in an *α*-minimal essential medium (*α*-MEM; Gibco BRL, Gaithersburg, MD, USA) supplemented with 10% fetal bovine serum (FBS; Hangzhou Sijiqing Biological Engineering Materials Co. Ltd., Zhejiang, China), 0.292 mg/mL L-glutamine (Invitrogen Life Technology, Carlsbad, CA, USA), 100 units/mL penicillin (Invitrogen), and 100 mg/mL streptomycin (Invitrogen) at 37°C under 5% CO_2_. Cells were cultured about 2 weeks and the medium was changed after every three days. We used BMMSCs and JMMSCs at passages 2-5 (P2-P5) in this study. We further identified the capacity of proliferation of these MSCs by MTT assay (ATCC, Manassas, VA, USA). The MSC positive markers CD105, CD73, and CD90 or negative markers, CD14, CD19, HLA-DR, CD34, and CD45 (BD Biosciences, San Diego, CA, USA), were measured using flow cytometric analysis. The capacity for multipotent differentiation, including osteogenic and adipogenic differentiation, was detected by alizarin red staining and western blotting for Runx2, SP7 (Santa Cruz Biotechnology, Dallas, Texas, USA), COL-1, and ALP (Abcam, Cambridge, UK) and by Oil Red O staining and western blotting for PPAR-*γ* and LPL (Abcam, Cambridge, UK).

Human monocytes were isolated from the peripheral blood of normal human volunteers (blood donors from the Blood Transfusion Department of Xijing Hospital) using a Human Monocyte Isolation Kit II (Miltenyi Biotec, Teterow, Germany). In brief, peripheral blood mononuclear cells were collected by density gradient separation using a Lymphocyte Separation Medium (TBD Science Biotech Company, Tianjin, China). Red blood cells were lysed by incubating cells in a red blood cell lysis buffer (BioFlux, Beijing, China) for 3 min, and mononuclear cells were washed with PBS. Then, cell pellets were resuspended and incubated with anti-human CD14 antibody (eBiosciences, San Diego, CA, USA) for 10 min and biotin-labeled microbeads (Miltenyi Biotec, Teterow, Germany) for 15 min at 4°C degree. Purified CD14^+^ monocytes were plated into 6-well cell culture plates at a concentration of 0.5‐1 × 10^6^ per well in RPMI 1640 media supplemented with 10% fetal bovine serum (FBS; Hangzhou Sijiqing Biological Engineering Materials Co. Ltd. Zhejiang, China).

### 2.3. Isolation and Characterization of Exosomes

To avoid contamination of serum exosomes, cells were cultured in a complete medium depleted of FBS-derived exosomes by ultracentrifugation at 100,000 g for 3 h at 4°C. Ten milliliters of culture supernatant was collected to isolate exosomes with ExoQuick-TC (ExoQuick; System Biosciences), according to manufacturers' protocol. Briefly, the supernatant was centrifuged at 3000 g for 15 min, mixed with 2 mL ExoQuick-TC exosome precipitation solution, and incubated for over 12 h at 4°C. Then, the mixture was centrifuged at 1500 g for 30 min. The samples were then loaded onto a carbon-coated electron microscopy grid and stained with sodium phosphotungstate for 30 s and air-dried and then were observed using transmission electron microscopy (HT7800, Hitachi, Japan). The exosome markers CD63 and CD81 were analyzed by using western blot. Moreover, the size of exosomes was measured by nanoparticle tracking analysis (NTA) (RiboBio Ltd., Guangzhou, China).

### 2.4. Skin Wound-Healing Model and Treatment

Skin-defective mice were established (*n* = 6) as previously described [15]. Briefly, following anesthesia and hair shaving at the dorsal surface, a 1.2 cm diameter full-thickness skin excision was created on the back of the mice. Meanwhile, the mice were randomly divided into group A (BMMSC group, injection, 2 × 10^6^ cells/mL), group B (JMMSC group, injection, 2 × 10^6^ cells/mL), and the control group C (phosphate-buffered saline (PBS) group, injection, 200 *μ*L). Then, we established the macrophage-depleted mice model (M-) (*n* = 6) through CL (clodronate liposomes, Nico van Rooijen lab, Holland) intravenous administration (5 mg/mL, 200 *μ*L), then injected MSCs after 48 h and randomly divided them into group D (BMMSC (M-) group, injection, 2 × 10^6^ cells/mL) and group E (PBS (M-) group, injection, 200 *μ*L). In order to maintain macrophage depletion, the CL was injected after every three days. In the exosome treatment experiment, skin-defective mice were established (*n* = 4) and randomly divided into group A (PBS group, injection, 200 *μ*L), group B (BMMSC group, injection, 2 × 10^6^ cells/mL), group C (BMSC-derived exosomes, injection, 200 *μ*g), and group D (siRab27a interfered BMMSCs, injection, 2 × 10^6^ cells/mL). Wound area was observed daily, and the wound-healing rate was calculated at different time points (days 3, 6, 9, and 12). The schemes for the description of the *in vivo* study are shown in Supplementary [Supplementary-material supplementary-material-1]. Wound areas were measured by tracing the wound margin and calculated using an image analysis program (ImageJ 1.48, National Institutes of Health). After the sacrifice of mice at the indicated time points, wound bed biopsies were divided into two parts for paraffin-embedded and frozen sections.

For MSCs labeled with CM-DIL (Thermo Fisher Scientific, Waltham, MA USA), which is a fluorescent dye well suited for monitoring cell movement or location and injected into the vein of the tail to explore the target cells of “homing” MSCs in the wound area, the adjacent normal skin was used as the control (*n* = 3). Mice were sacrificed on day 7 after treatment and skin samples were harvested for further analysis.

### 2.5. Histological and Immunohistochemistry Staining

The wound skin and surrounding skin were fixed in 4% paraformaldehyde, embedded in paraffin, and cut into 4 *μ*m sections. Standard HE staining and Masson trichrome staining were performed. To investigate the polarization of M2 macrophages *in vivo* and *in vitro*, indirect immunofluorescence studies of CD68 (sc-9139, 1 : 200), resistin-like molecule- (RELM-) *α* (sc-16120, 1 : 200), and CD14 (sc-9150, 1 : 200) and CD163 (sc-18796, 1 : 200, Santa Cruz Biotechnology, Dallas, Texas, USA) were performed as previously described [4]. Immunohistochemical analysis for CD31 (Abcam, ab28364, 1 : 20) and PCNA (Abcam, ab2426, 1 : 200) was performed as previously described [16]. The secondary antibodies, including donkey anti-rabbit IgG-FITC, Alexa Fluor 594 AffiniPure Donkey Anti-Goat IgG (H+L), and Peroxidase AffiniPure Goat Anti-Rabbit IgG (H+L), were purchased from Jackson ImmunoResearch Laboratories. For semiquantification, positive signals from at least five random high-power fields were visualized, counted, and expressed as a percentage of total DAPI-positive cells (mean ± SD).

### 2.6. Coculture of MSCs or MSC-Derived Exosomes with Macrophages

For coculture studies, the CD14-positive monocytes were seeded into 6-well plates in RPMI 1640 media supplemented with 10% FBS, and on day 7, 2 × 10^5^ BMMSCs or JMMSCs were seeded into the 0.4 *μ*m pore size Transwell inserts (Millipore, Darmstadt, Germany) in an *α*-MEM containing 10% FBS, cocultured with macrophages for another 3 days. To study the function of MSC-derived exosomes on macrophage polarization, 2 × 10^5^ BMMSCs, 50 *μ*g/mL MSC-derived exosomes, and 2 × 10^5^ BMMSCs transfected with siRab27a for 48 h were seeded with human PBMC-derived macrophages on day 7 and cultured for another 3 days. Then, macrophages were processed for the flow cytometric analysis (CytoFLEX, Beckman Coulter, CA, USA) of cell surface marker CD206 (BioLegend, San Diego, CA, USA), and the expression of CD206 was analyzed (R&D Systems, Minneapolis, Minnesota, USA) using immunofluorescence and RT-PCR.

### 2.7. Macrophages Uptake MSC-Derived Exosomes

MSC-derived exosomes were labeled with PKH26 (Sigma-Aldrich, St. Louis, MO, USA), as previously described with minor modification [17]. Human peripheral blood PBMC-derived macrophages, on day 7, were previously cultured with PKH26-labeled exosomes for 24 h at 37°C under 5% CO_2_. After incubation, macrophages were washed twice with PBS and fixed in 4% paraformaldehyde for 20 min at room temperature. The sample was then washed twice with PBS and labeled with 4′,6-diamidino-2-phenylindole (DAPI; Vector Laboratories, Burlingame, CA). Macrophage uptake of MSC-derived exosomes was observed under confocal laser microscope (Zeiss, Oberkochen FV1000, Germany).

### 2.8. Small Interfering RNA and Transfection Assays

For siRNA inhibition studies, MSCs were grown to 60% confluence followed by serum starvation for 12 h. siRab27a and a negative control (Santa Cruz Biotechnology, Dallas, Texas, USA) were transfected into cells at a final concentration of 50 nM using the Lipo2000 (Invitrogen, Carlsbad, CA, USA) according to the manufacturer's instructions. After transfection, the cells were harvested at 48 h for protein extraction.

For microRNA studies, MSCs were transfected with the miR-223 mimic at a final concentration of 50 nM and miR-223 inhibitor at a final concentration of 100 nM using the Lipo2000 (Invitrogen, Carlsbad, CA, USA) according to the manufacturer's instructions. After transfection, supernatant fractions collected from 48-hour cultures were used to isolate exosomes.

### 2.9. Western Blot Analysis

Cell lysates or mice skin homogenates were extracted using lysis buffer (10 mM Tris–HCl, 1 mM ethylene diamine tetra acetic acid (EDTA), 1% sodium dodecyl sulfate, 1% Nonidet P-40, 1 : 100 proteinase inhibitor cocktail, and 50 mM *β*-glycerophosphate, and 50 mM sodium fluoride) (Beyotime, Shanghai, China). The protein concentration was determined with a protein assay kit (Beyotime, Shanghai, China), following the manufacturer's instructions. Aliquots of 40 to 50 *μ*g per sample were separated by 10% sodium dodecyl sulfate-polyacrylamide gel electrophoresis (SDS-PAGE), transferred to polyvinylidene fluoride (PVDF) membranes (Millipore, Billerica, MA, USA), and blocked with 5% bovine serum albumin (BSA) in PBST (PBS with 0.1% Tween). Then, they were incubated with the following primary antibodies overnight: RELM-*α* (Santa Cruz Biotechnology, Dallas, Texas, USA), CD63 (Santa Cruz Biotechnology, Dallas, Texas, USA), CD81 (Santa Cruz Biotechnology, Dallas, Texas, USA), Rab27a (Abcam, Cambridge, UK), pknox1 (Abcam, Cambridge, UK), and anti-GAPDH (Abcam, Cambridge, UK). Then, the membranes were incubated with a horseradish peroxidase-conjugated secondary antibody (Boster, Wuhan, China). The blots were visualized using an enhanced chemiluminescence kit (Amersham Biosciences, Piscataway, NJ, USA), according to the manufacturer's instructions.

### 2.10. Total RNA Extraction and Quantitative RT-PCR

Total cellular RNA was extracted using a TRIzol reagent (Invitrogen, Carlsbad, CA, USA), according to the manufacturer's instructions. Isolated total RNA was then subjected to reverse transcription using Oligo dT primer and PrimeScript® RTase (Takara, Dalian, China), according to the manufacturer's instructions. Quantitative RT-PCR (qRT-PCR) was performed with SYBR® Premix Ex Taq™ II (Takara, Dalian, China) using the C1000™ Thermal Cycler (Bio-Rad, Hercules, CA, USA). The expression levels of the target genes were normalized to that of the housekeeping gene, GAPDH. The sequences of primers used are shown in Supplementary [Supplementary-material supplementary-material-1].

### 2.11. Statistical Analysis

All *in vitro* experiments were performed in triplicates with three different groups. The values were shown as the mean ± standard deviation (SD). The statistical differences between two groups were determined using two-tailed unpaired Student's *t*-test while those for more than two groups were determined using one-way Analysis of Variance (ANOVA) with the Bonferroni correction. All statistical analyses were done using GraphPad Prism 5.0 (GraphPad Software, La Jolla, CA, USA), and *P* values less than 0.05 were considered statistically significant.

## 3. Results

### 3.1. MSC-Based Therapy Is Macrophage-Dependent and Promotes Cutaneous Wound Healing

**Figure 1:**
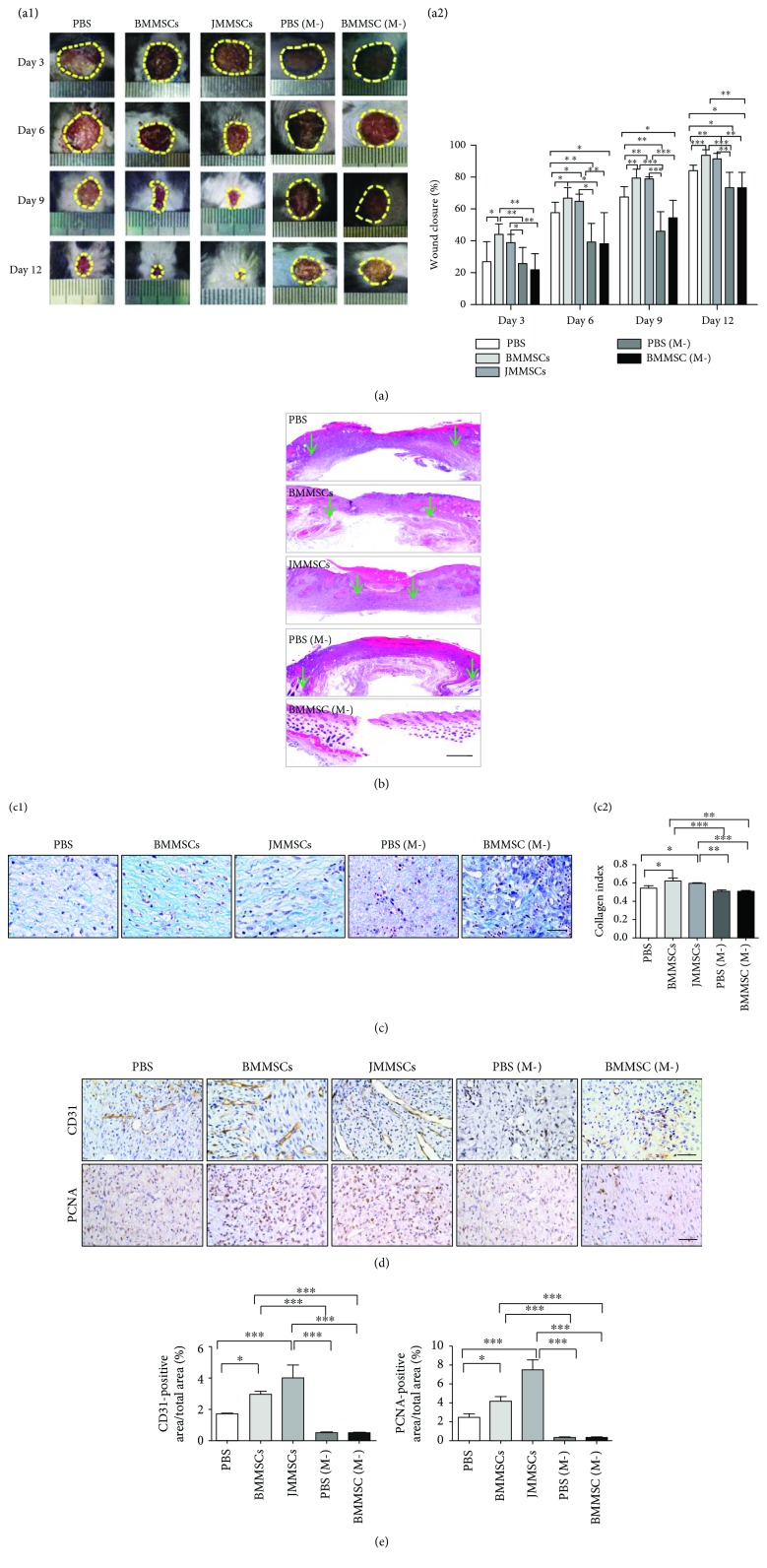
MSC-based therapy is macrophage-dependent and enhances cutaneous wound healing. (a) Representative light field photographs of cutaneous wounds in normal mice or macrophage-depleted mice after treatment with PBS, BMMSCs or JMMSCs (a1); the whole cutaneous wound is outlined in a dashed line. Percentage of the wound closure on day 3 to day 12 in reference to the day 0 wounds from the groups described in the left figures (a2) (*n* = 6). (b) Representative H&E image from a cutaneous wound at day 12, the green arrows indicating the wound edge. (c) Masson trichrome (c1) showing collagen deposition at day 12 and quantification of collagen index (c2) (*n* = 3). (d) Immunostaining of CD31 and proliferating cell nuclear antigen (PCNA) at day 12 of skin wound (*n* = 3). (e) Quantification of immunostaining of CD31 and PCNA positively stained area percentages at day 12 of skin wound (*n* = 3). Scale bars: 500 *μ*m (b) and 50 *μ*m (c, d). ^∗^*P* < 0.05, ^∗∗^*P* < 0.01, and ^∗∗∗^*P* < 0.001. Error bars are mean ± SD.

BMMSCs and JMMSCs were characterized for surface markers, osteogenesis, and adipogenesis (Supplementary [Supplementary-material supplementary-material-1]). To investigate the roles of MSCs in wound healing, two kinds of MSCs were systemically infused into mice 1 day post full-thickness skin excision and wound closure was carefully assessed after every three days (*n* = 6). Our results showed that mice that were infused with both kinds of MSCs exhibited accelerated skin wound closure compared with the control mice infused with only PBS (Figures 1(a) and 1(b)). The enhancement in wound closure appeared on day 3, and the wound became completely closed on day 12, which was as efficient as that shown by a previous study on the effect of gingiva-derived MSCs on promoting wound closure [4]. Moreover, to determine the role of macrophages in MSC-based therapy, we depleted macrophages in the early stage (Supplementary [Supplementary-material supplementary-material-1]) and observed the wound closure of mice with and without BMMSC therapy. Early depletion of macrophages significantly delayed the wound closure compared with that in the PBS, BMMSC infusion, and JMMSC infusion groups (Figures 1(a) and 1(b)), which indicated that macrophages were required in wound healing and that MSC therapy did not rescue the phenotype induced by macrophage depletion.

Collagen formation was evaluated in the form of a collagen index according to the previous report [18]. Masson trichrome staining showed a higher degree of collagen formation in the BMMSC and JMMSC treatment groups than in the PBS group and the macrophage depletion groups (Figure 1(c)). In addition, to further determine the effects of MSC on wound healing, we stained the vascular endothelial marker, CD31, and proliferative marker, PCNA, in the wound bed area. We discovered that the percentage of the CD31 and PCNA positively stained area increased upon BMMSC or JMMSC treatment as compared with that in the PBS group and the macrophage depletion groups (Figures 1(d) and 1(e)). These findings indicated that BMMSC or JMMSC treatment might lead to more prominent effects on angiogenesis and cell proliferation during wound healing.

### 3.2. Systemically Infused MSCs Home to the Wound Site and Skew Macrophages to M2

In order to investigate the *in vivo* interaction of MSCs and host macrophages, BMMSCs and JMMSCs, prelabeled with CM-DiL, were systemically injected into mice (*n* = 3). The numbers of BMMSCs and JMMSCs that home to the wound site were increased compared with the cell numbers in the same site of normal skin (Supplementary [Supplementary-material supplementary-material-1]). However, there was no significant differences between the numbers of BMMSCs and JMMSCs that were homing to the wound site (Supplementary [Supplementary-material supplementary-material-1]). In addition, BMMSCs and JMMSCs were in close proximity with CD68-positive macrophages at the wound site (Supplementary [Supplementary-material supplementary-material-1]).

**Figure 2:**
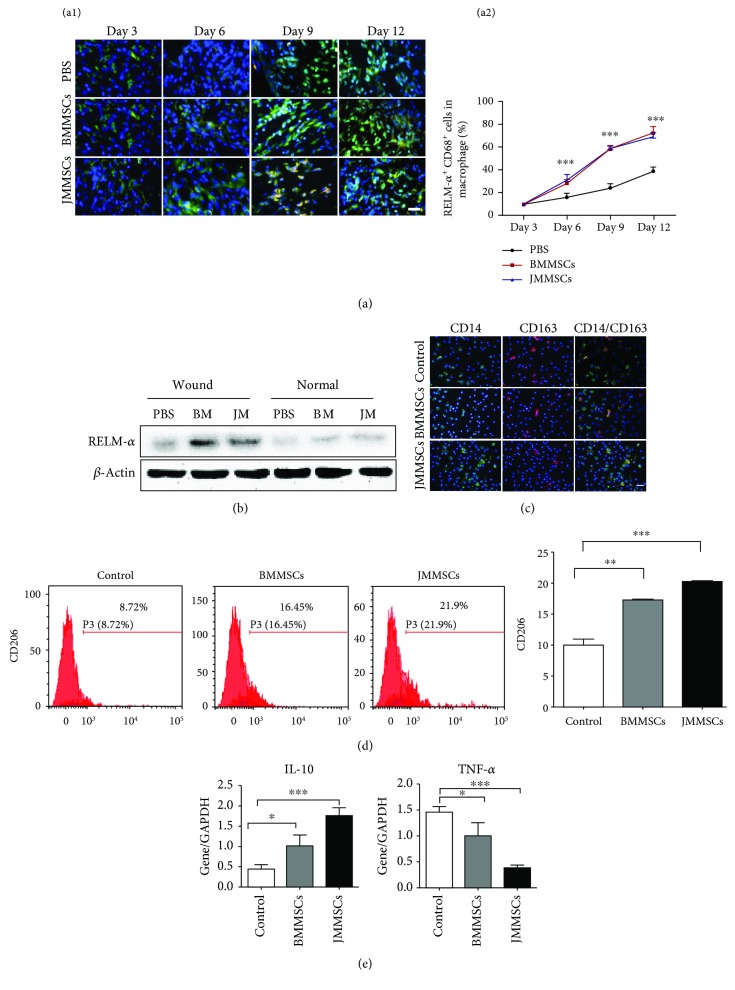
Systemically infused MSCs skew macrophages to M2. (a) Dual-color immunofluorescence staining of CD68 (green) and RELM-*α* (red) at the wound site after systemic injection of BMMSCs and JMMSCs at days 3, 6, 9, and 12 (a1). Cell nuclei were counterstained with DAPI (blue). Comparison of the percentage of RELM-*α* and CD68 dual-positive macrophages (a2) (*n* = 3). (b) Western blot analysis of RELM-*α* expression in the wound samples and the surrounding normal skin samples after systemic injection of BMMSCs and JMMSCs. (c) Dual-color immunofluorescence staining of CD14 (green) and CD163 (red) in macrophages after being cocultured with BMMSCs or JBMMSCs. (d) The percentage of CD206-positive cells in macrophages after coculture with BMMSCs or JMMSCs by flow cytometry (*n* = 3). (e) qRT-PCR analysis of IL-10 and TNF-*α* in macrophages after being cocultured with BMMSCs or JBMMSCs (*n* = 3). Scale bars: 100 *μ*m (a, c). ^∗^*P* < 0.05, ^∗∗^*P* < 0.01, and ^∗∗∗^*P* < 0.001. Error bars are mean ± SD.

We next explored the *in vivo* effects of BMMSCs and JMMSCs on the phenotype of macrophages located at the wound area. Macrophages were stained using dual-color immunofluorescence, specific antibodies for CD68 (green), and RELM-*α* (red). CD68 is a surface marker of macrophage [19, 20]. RELM-*α* is a well-known marker for M2 macrophages [21, 22] and macrophages showing the wound-healing phenotype [23]. After infusion of BMMSCs and JBMMSCs, the time-dependent increase in the number of both RELM-*α* and CD68-positive cells (yellow) was observed (Figure 2(a)). We also verified that systemic injection of BMMSCs and JMMSCs could promote the RELM-*α* expression at the wound site, but not in the normal skin (Figure 2(b)).

To further investigate whether BMMSCs and JMMSCs convert macrophages into those with the M2 phenotype, human PBMC-derived macrophages were, respectively, cocultured with BMMSCs or JMMSCs at a ratio 1 : 2.5~1 : 5 for 72 h in the Transwell system. Then, macrophages were stained with CD14 and CD163, which is an M2a marker, induced by IL-4 or IL-13 and associated with tissue repair [24]. The results showed a higher number of CD14 and CD163 double-positive macrophages (Figure 2(c)) after coculturing with MSCs. In addition, the expression of CD206, a marker of an M2 and wound-healing macrophage [23], and HLA-DR [25], one of the markers for M1 macrophages, was assessed in CD14^+^ macrophages after coculturing with BMMSCs or JMMSCs using flow cytometry. The results showed a higher number of CD206 macrophages in BMMSC or JMMSC group compared to the control group (Figure 2(d), Supplementary [Supplementary-material supplementary-material-1]). However, there was no significant difference in expression of HLA-DR among the three groups (Supplementary [Supplementary-material supplementary-material-1]). The macrophages also expressed higher levels of IL-10 and lower levels of TNF-*α* after coculturing with BMMSCs or JBMMSCs compared to the control group (Figure 2(e)). Taken together, these results elucidated the positive effects of BMMSCs or JMMSCs in inducing M2 polarization of macrophages both *in vivo* and *in vitro*.

### 3.3. Uptake of MSC-Secreted Exosomes by Macrophages Promotes M2 Polarization

**Figure 3:**
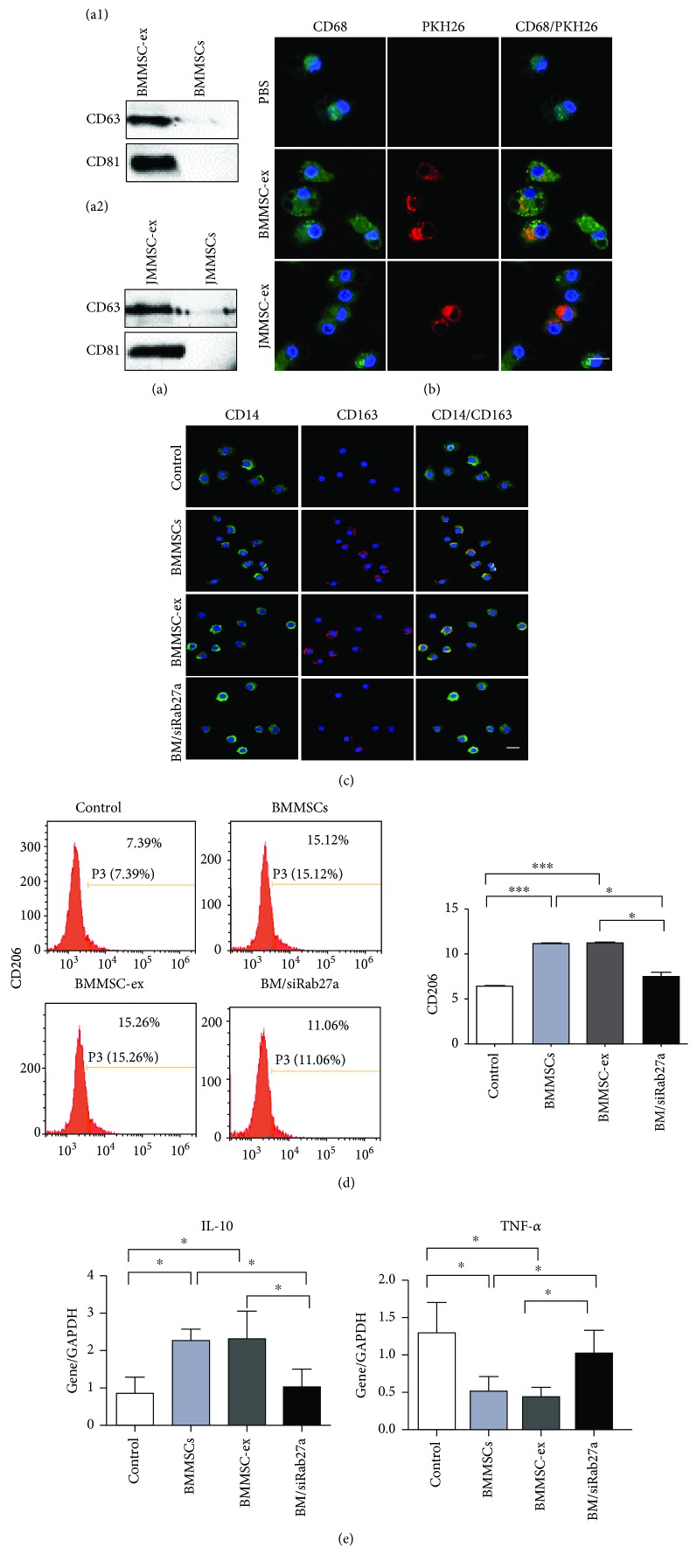
Uptake of MSC-secreted exosomes by macrophages promotes M2 polarization. (a) The expression of CD63 and CD81 in BMMSC-ex and BMMSCs (a1) and JMMSC-ex and JMMSCs (a2) assessed by western blot analysis. (b) Exosomes (PKH26, red) from BMMSCs or JBMMSCs entered into macrophages (CD68, green). (c) CD14 (green) and CD163 (red) staining of macrophages after being cocultured with BMMSC-, BMMSC/siRab27a-, or BMMSC-derived exosomes. Macrophages cocultured with BM/siRab27a showed less CD14 and CD163 double-positive cells compared to the BMMSC or BMMSC-ex group. (d) CD206-positive macrophages after being cocultured with exosomes and BMMSCs were assessed by flow cytometric analysis (*n* = 3). CD206 expression increased compared with the macrophages without treatment. However, the number of CD206-positive macrophages decreased after coculture with BMMSCs of Rab27a knockdown compared to the BMMSC and BMMSC-ex groups. (e) qRT-PCR analysis of IL-10 and TNF-*α* in macrophages after being cocultured with BMMSC-, BMMSC/siRab27a-, or BMMSC-derived exosomes (*n* = 3). Macrophages cocultured with BM/siRab27a showed lower IL-10 and higher TNF-*α* compared to the BMMSC or BMMSC-ex group. Scale bars: 50 *μ*m (b, c). ^∗^*P* < 0.05 and ^∗∗∗^*P* < 0.001. Error bars are mean ± SD.

These findings led us to investigate which factors participate in MSC-induced polarization of M2 macrophages. Next, we isolated exosomes secreted by BMMSCs (BMMSC-ex) or JMMSCs (JMMSC-ex) and observed them using transmission electron microscopy (TEM). Exosomes exhibited a cup-shaped morphology, as shown by TEM (Supplementary [Supplementary-material supplementary-material-1]). Nanoparticle tracking analysis (NTA) revealed that the isolated exosomes from BMMSCs possessed diameters ranging from 20 to 200 nm, with a mean diameter of 27.46 nm (Supplementary [Supplementary-material supplementary-material-1]), and the exosomal markers, such as CD63 and CD81, were examined in BMMSC-ex and JMMSC-ex (Figure 3(a)). We added the PKH26-labeled BMMSC-ex or JMMSC-ex into the macrophage cultures, and after 24 h, the PKH26-labeled exosomes were observed in macrophages. However, PBS group cells did not exhibit any red fluorescence (Figure 3(b)). We also collected the supernatant of BMMSCs and JMMSCs and measured the total amount of exosome protein purified from culture medium (Supplementary [Supplementary-material supplementary-material-1]). Western blot analysis also showed that BMMSCs and JMMSCs expressed Rab27a (Supplementary [Supplementary-material supplementary-material-1]), which regulated the release of exosomes [25].

To know whether exosomes are involved in BMMSC-mediated polarization of M2 macrophages, we used Rab27a siRNA to decrease exosome secretion of BMMSCs. Firstly, the expression of Rab27a was downregulated after BMMSCs were transfected with Rab27a siRNA (BM/siRab27a, Supplementary [Supplementary-material supplementary-material-1]). Then, the exosome secretion was inhibited after Rab27a knockdown (Supplementary [Supplementary-material supplementary-material-1]). After that, BMMSCs, BMMSC-ex, and BM/siRab27a were added to the culture medium of macrophages. Macrophages without coculture were used as the control. The results showed that the percentage of CD206-positive cells was increased in the three groups after coculturing with BMMSCs, BMMSC-ex, and BM/siRab27a compared to the control group (Figure 3(d)). However, BM/siRab27a decreased the M2 polarization of macrophages compared to the BMMSC and BMMSC-ex groups (Figure 3(d)). The immunofluorescence staining of CD14 and CD163 showed a higher number of CD14 and CD163 double-positive cells after coculturing with BMMSCs or BMMSC-ex compared to the control group (Figure 3(c)). Macrophages cocultured with BM/siRab27a showed a lower number of CD14 and CD163 double-positive cells compared to the BMMSC or BMMSC-ex groups (Figure 3(c)). Compared with the control group, macrophages expressed a higher level of IL-10 and a lower level of TNF-*α* after coculturing with BMMSCs or BMMSC-ex. However, there was no significant difference in the expression of either IL-10 or TNF-*α* in macrophages cocultured with BM/siRab27a (Figure 3(e)).

### 3.4. MSCT Enhances Cutaneous Wound Healing and Skews Macrophages to the M2 Phenotype through Exosomes

**Figure 4:**
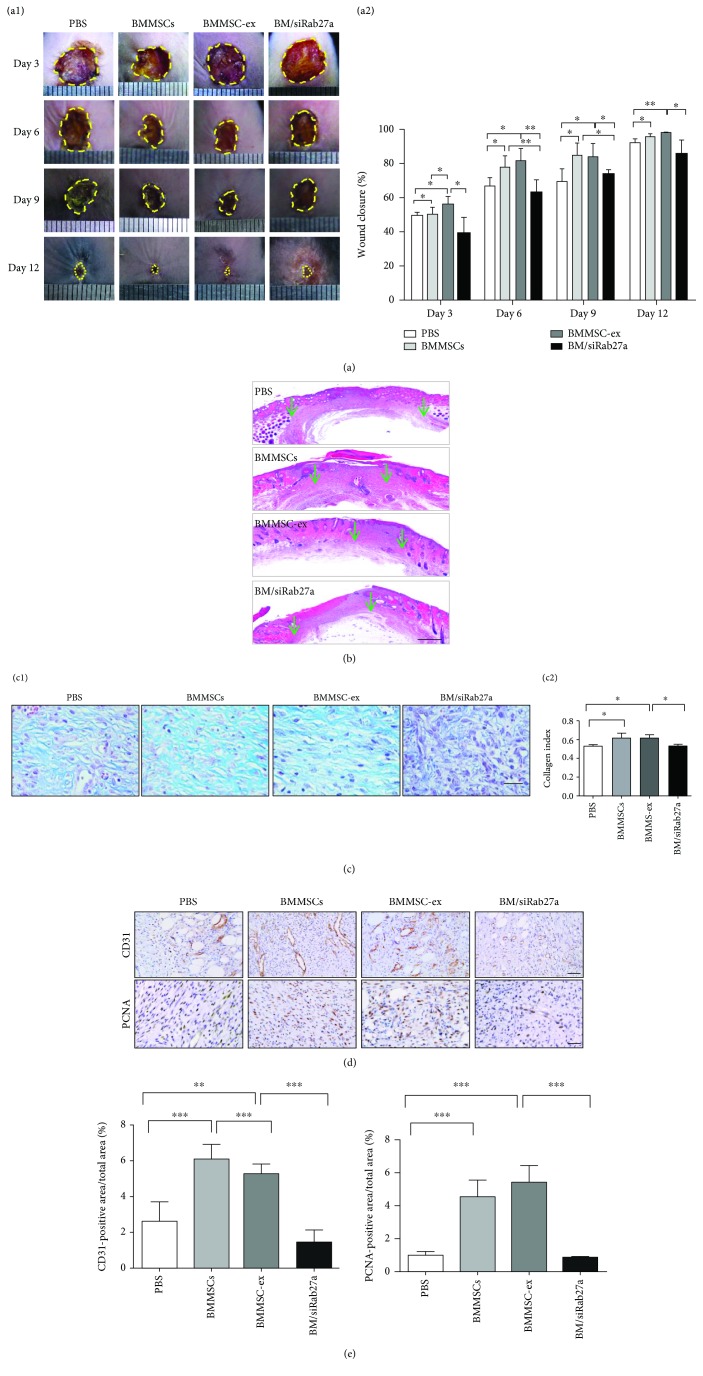
MSCs enhance wound healing through exosomes. (a) Representative light field photographs of cutaneous wounds after treatment with PBS, BMMSCs, BMMSC-ex, and BM/siRab27a (a1); the whole cutaneous wound is outlined in a dashed line. Percentage of the wound closure on day 3 to day 12 in reference to the day 0 wounds (a2) (*n* = 4). BMMSC- and BMMSC-ex-treated wounds showed a statistically significant increase in wound closure comparing with the PBS-treated wounds, whereas BM/siRab27a-treated wounds had no significant difference comparing with the PBS group at different time points. (b) Representative H&E image from a cutaneous wound at day 12, the green arrows indicating the margin of wound-healing area. (c) Masson trichrome (c1) showing collagen deposition at day 12 and quantification of collagen index (c2) (*n* = 3). (d) CD31 and PCNA staining of the skin wound at day 12. (e) Quantification of immunostaining of CD31- and PCNA-positive cells at day 12 of the skin wound (*n* = 3). Scale bars: 500 *μ*m (b) and 50 *μ*m (c, d). ^∗^*P* < 0.05, ^∗∗^*P* < 0.01, and ^∗∗∗^*P* < 0.001. Error bars are mean ± SD.

Next, we investigated the *in vivo* effects of exosomes secreted by BMMSCs on wound repair and M2 polarization. BMMSCs, BMMSC-ex, and BM/siRab27a were systemically infused into mice 1 day post full-thickness skin excision, and wound closure was carefully assessed after every three days (*n* = 4). As shown, the mice that received BMMSC and BMMSC-ex infusion had substantially accelerated cutaneous wound healing, while BM/siRab27a infusion delayed wound healing at days 3, 6, 9, and 12 (Figures 4(a) and 4(b)). Masson trichrome staining also showed a higher degree of collagen formation in the BMMSC or BMMSC-ex treatment groups compared to the PBS and BM/siRab27a groups (Figure 4(c)). In addition, a higher proportion of the CD31 and PCNA positively stained area was observed in BMMSC- or BMMSC-ex-treated wounds as compared with the PBS and BM/siRab27a groups (Figures 4(d) and 4(e)). These results demonstrated the promoting effects of BMMSC-derived exosomes on cutaneous wound healing.

**Figure 5:**
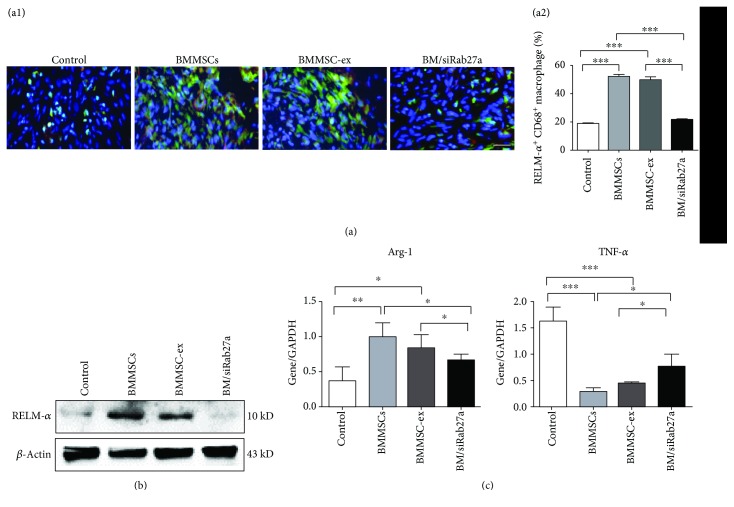
MSCs skew macrophages to M2 through exosomes. (a) Immunofluorescence staining of CD68 (green) and RELM-*α* (red) at the wound site after systemic injection of BMMSC-, BMMSC/siRab27a-, or BMMSC-derived exosomes (a1); the percentage of M2 in CD68^+^ macrophages (a2) (*n* = 3). (b) Western blot analysis of RELM-*α* expression at the wound site. RELM-*α* expression was increased in the BMMSC and BMMSC-ex groups compared to the PBS and BM/siRab27a groups. (c) qRT-PCR analysis of Arg-1and TNF-*α* at the wound site. A high level of TNF-*α* and a low level of Arg-1 were detected in the PBS and BM/siRab27a groups compared to the BMMSC and BMMSC-ex groups (*n* = 3). Scale bar: 100 *μ*m (a).^∗^*P* < 0.05, ^∗∗^*P* < 0.01, and ^∗∗∗^*P* < 0.001. Error bars are mean ± SD.

Further analysis on the CD68 and RELM-*α* double-positive cells at the wound site confirmed the positive roles of exosomes on the M2 polarization of macrophages. The results showed that the number of CD68 and RELM-*α* double-positive cells were increased in the BMMSC and BMMSC-ex groups compared to the PBS and BM/siRab27a groups (Figure 5(a)). In addition, western blot assay of the wound site tissue showed similar effects, in that the expression of RELM-*α* was increased in the BMMSC and BMMSC-ex groups (Figure 5(b)). Moreover, expression of Arg-1 in the wound site was increased and expression of TNF-*α* was decreased in the BMMSC and BMMSC-ex groups compared to the PBS and BM/siRab27a groups as shown by qRT-PCR analysis (Figure 5(c)).

### 3.5. MSCs Skew Macrophages to the M2 Phenotype via Transferring Exosome-Derived miR-223

**Figure 6:**
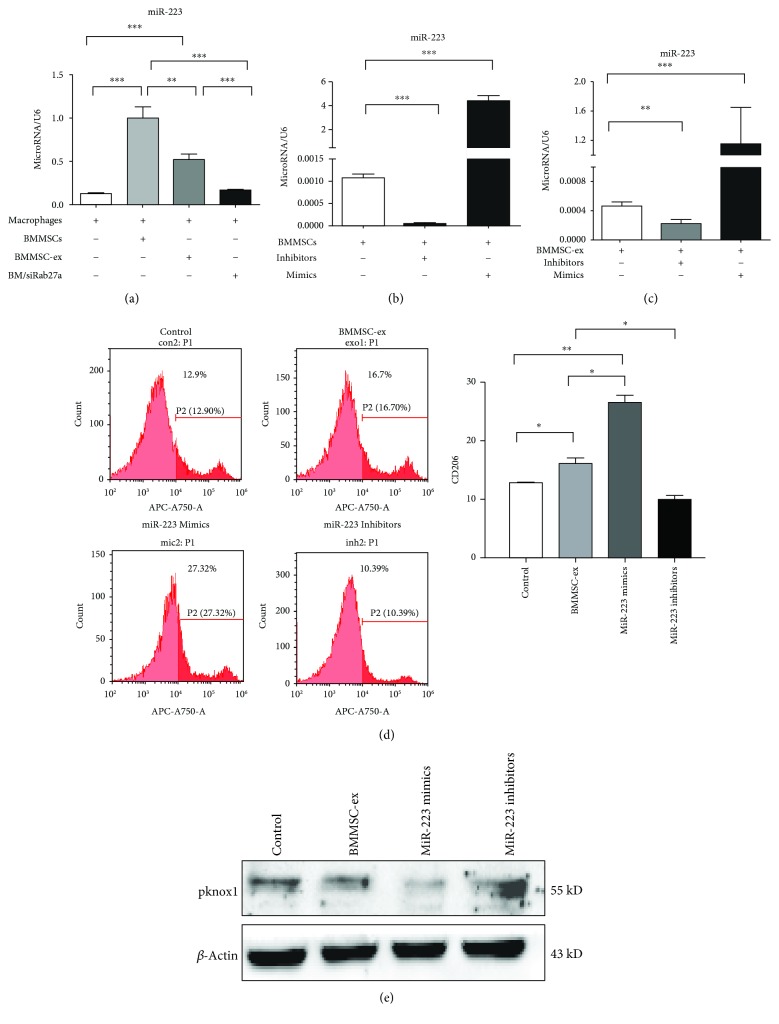
MSCs skew macrophages to M2 via transferring exosome-derived miR-223. (a) qRT-PCR analysis of miR-223 in macrophages cocultured with BMMSCs, BMMSC-ex, and BM/siRab27a. (b) Analysis of miR-223 in BMMSCs transfected with miR-223 mimics and inhibitors. (c) Analysis of miR-223 in exosomes derived from BMMSCs transfected with miR-223 mimics and inhibitors. (d) CD206-positive macrophages were assessed after being cocultured with exosomes derived from BMMSCs, which were transfected with miR-223 mimics or inhibitors (*n* = 3). (e) Western bolt analysis of pknox1 in macrophages after being cocultured with exosomes derived from BMMSCs, which were transfected with miR-223 mimics or inhibitors. ^∗^*P* < 0.05, ^∗∗^*P* < 0.01, and ^∗∗∗^*P* < 0.001. Error bars are mean ± SD.

miR-223 has been previously reported to promote macrophages to the M2 phenotype [26]. Collino et al. [27] reported that miR-223 was expressed in MSCs. Therefore, we first examined whether MSCs transferred miR-223 to macrophages. After coculturing with BMMSCs and BMMSC-ex, we investigated the expression of miR-223 in macrophages. The results showed that the expression of miR-223 was increased in macrophages cocultured with BMMSCs or BMMSC-ex compared to macrophages not cocultured (Figure 6(a)). Then, we used miR-223 mimics or inhibitors to overexpress or inhibit the miR-223 expression in BMMSCs, respectively (Figure 6(b)), and we investigated the expression of miR-223 in exosomes secreted by BMMSCs. miR-223 expression was profoundly inhibited and promoted after transfection with miR-223 inhibitors and mimics, respectively (Figure 6(c)). To determine whether miR-223 regulates M2 polarization of macrophages, we detected the CD206 expression of macrophages after culturing with exosomes, in which miR-223 was overexpressed or knocked down. Flow cytometry analysis showed a higher number of CD206-positive macrophages in the miR-223 mimic group and less number of CD206-positive macrophages in the miR-223 inhibitor group compared to those cultured with exosomes without treatment (Figure 6(d)). Considering pknox1 is a validated target gene of miR-223, we detected whether miR-223 in exosomes suppresses the pknox1 protein level in macrophages after coculturing. As anticipated, western blot assays revealed that overexpression of miR-223 significantly diminished accumulation of the pknox1 protein, whereas knockdown of miR-223 elevated pknox1 protein levels (Figure 6(e)). Taken together, these results showed that exosome-derived miR-223 may be an important factor to promote macrophages to the M2 phenotype.

## 4. Discussion

During the wound-healing process, immune cells reside in the wound site where they regulate inflammation and mediate the tissue repair [28]. Despite entrapment of intravenously injected MSCs in the lung, they are still capable of migrating to the site of inflammation and injury [29]. MSCs exert their immunomodulatory properties by regulating the function of both innate and adaptive immune cells via mechanisms involving both direct cell-cell contact and/or soluble factors [12, 13, 30, 31]. MSCs can play an important role in the wound-healing process via the secretion of soluble factors, such as TGF-*β*1 [32] and TSP-1 [33]. However, cell-cell interaction after MSCT that promotes skin repair still remains unclear.

The inflammatory response is a crucial component of cutaneous wound healing, as evidenced by severely delayed repair following *in vivo* macrophage ablation [6]. In response to signals derived from the injury, macrophages undergo a reprogramming that leads to the emergence of a spectrum of distinct functional phenotypes. Depending on the cytokines IFN-*γ* and TNF-*α*, M1 macrophages upregulate the enzyme-inducible nitric oxide synthase (iNOS) and produce a variety of proinflammatory cytokines, including IL-1, IL-6, and IL-23. Conversely, M2 macrophages, dependent on IL-4 and IL-13, released from T_H_2 lymphocytes in response to tissue injury, upregulate the enzymes Arginase 1, Fizz, and Ym1 [34]. A study by Chen et al. [35] found that MSCs can promote macrophage M2 polarization by secreting TGF-*β*3 and TSP1. Growing evidence has shown that M2 macrophages resolve the inflammation and promote wound healing [7, 8]. Human gingiva-derived MSC transplantation enhanced cutaneous wound healing by inducing M2 polarization of macrophages at the wound site [4]. In our study, we also found that MSCT promotes M2 polarization of macrophages at the wound site. The expression of M2-specific factors, such as RELM-*α* and Arginase 1, was increased at the wound site. Furthermore, MSCs also induced M2 macrophage differentiation *in vitro*. The macrophages expressed higher levels of IL-10 and lower levels of TNF-*α* after coculturing.

Exosomes contain several molecules, such as proteins and miRNAs, and serve as a new mechanism for cell-cell communication [36, 37]. Tumor-derived exosomes are important tumorigenesis mediators capable of inducing neoplastic transformation and tumor metastasis in stromal/stem cells [38, 39]. Meanwhile, stromal cell-derived exosomes promote cancer cell migration [40]. These evidences suggested that exosomes mediate the crosstalk between tumor cells and surrounding stromal cells. Recently, increasing amount of evidence of the therapeutic potential of MSC-derived exosomes in promoting cutaneous wounding healing has emerged [12, 41]. Exosomes derived from human umbilical cord MSCs enhance proliferation and migration of skin cells via Wnt4-mediated *β*-catenin nuclear translocation [12]. In our study, when we inhibited the secretion of exosomes in MSCs, the number of M2 macrophages was decreased both in the *in vitro* coculture system and in the *in vivo* interaction site. These results indicated that MSCT can elicit M2 polarization of macrophage by secreting exosomes.

Exosomes have been demonstrated to play an important role in skin wound healing; however, to our knowledge, only a few studies have reported the effects of MSC-derived exosomes on M2 polarization of the macrophage. Exosomes contain microRNA and are involved in intracellular communication. We revealed that exosomes secreted by MSCs contained miR-223, which contributed to macrophage polarization. miR-223, which suppresses classic proinflammatory pathways and enhances the alternative anti-inflammatory responses, is a novel regulator of macrophage polarization [26, 42, 43]. In addition, pknox1 is identified as a genuine miR-223 target gene and an essential regulator of macrophage polarization [44]. Here, we also found that knockdown of miR-223 in MSCs reduced M2 polarization of the macrophage. Alteration of the pknox1 expression was observed in the macrophage after coculturing with exosomes isolated from BMMSCs that were transfected with miR-223 mimics or inhibitors. Previous studies have showed that exosomes derived from LPS-preconditioned MSCs contained let-7b, which skewed M2 polarization of the macrophage [41, 45]. In addition, miR-146a has been reported to negatively regulate the wound healing in a diabetic murine wound-healing model [46]. We could not preclude the other miRNAs or factors contained in exosomes derived from MSCs that may induce M2 polarization during MSCT. MSCT may use multiple mechanisms to promote cutaneous wound healing, and further study is still needed to explore the other mechanisms of MSCT.

Taken together, our findings provided the evidence that MSCT elicits M2 polarization of macrophages and accelerates wound healing, in part, via transferring donor exosome-derived microRNA. Thus, the microRNAs of exosomes derived from MSCs could be a therapeutic target for cutaneous wound healing.

## Data Availability

The data used to support the findings of this study are included within the article.

## Supplementary Materials

Supplementary Materials The supplementary material file is the supplementary figures associated with the results that might be of interest to readers.
