# Supplementary material for: MSC-Derived Exosome Promotes M2 Polarization and Enhances Cutaneous Wound Healing
Source: Stem Cells Int. 2019 Sep 9;2019:7132708. doi: 10.1155/2019/7132708 (PMC6754952; doi:10.1155/2019/7132708)
Supplement: Supplementary Materials — The supplementary material file is the supplementary figures associated with the results that might be of interest to readers. [file 7132708.f1.pdf]

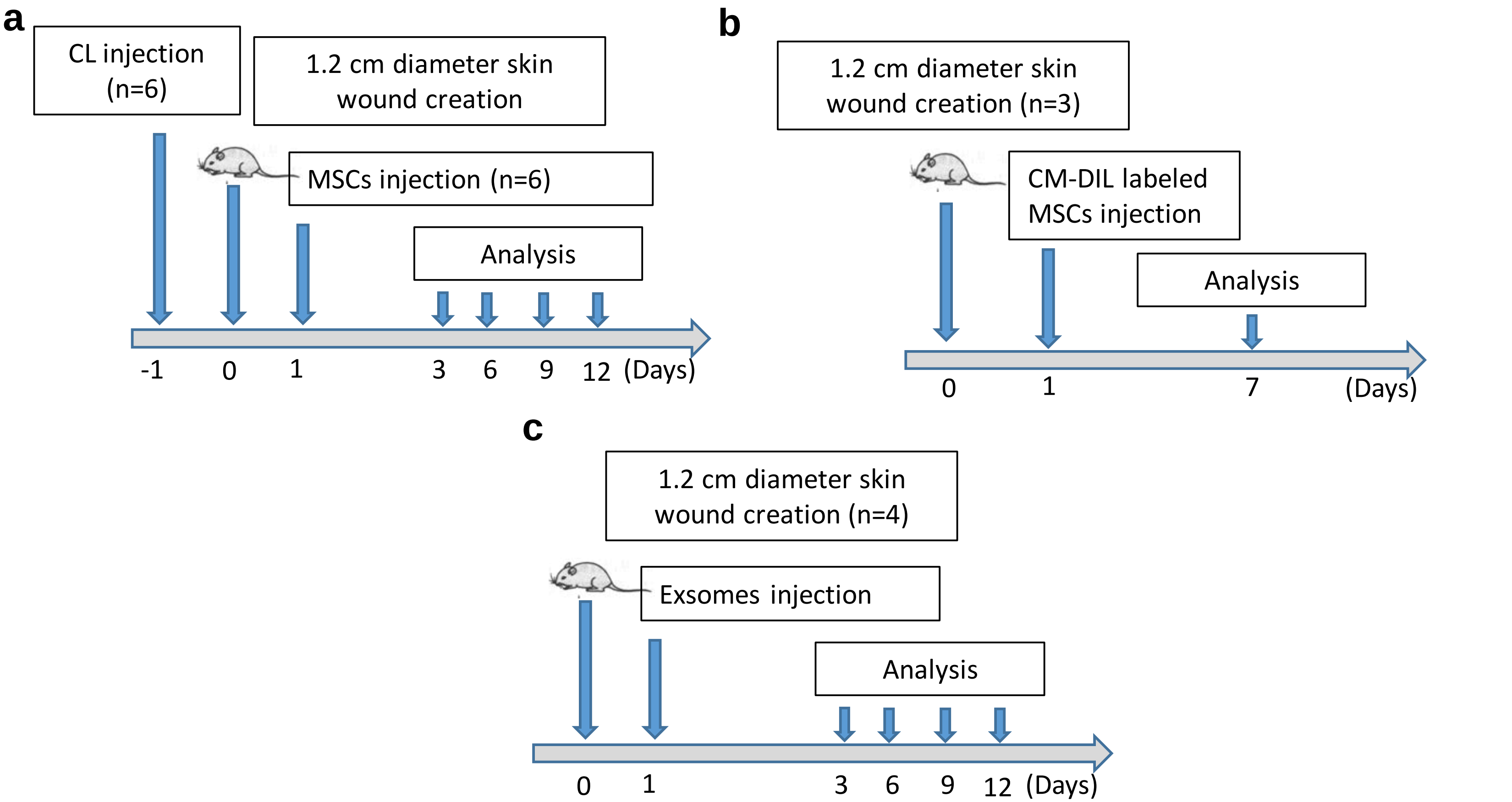

Supplementary Figure S1. The schemes for the description of the in vivo study. (a) Scheme illustrating the cutaneous wound procedure and treatment with CL and MSCs. Full-thickness excision cutaneous wounds (1.2 cm × 1.2 cm) were created in the mid-backs of mice. One day before wound creation, 12 mice were intravenously injected with CL (5 mg/ml, 200 μl per mouse), and divided into two groups (PBS (M-) and BMMSC(M-)). One day after wound creation, 18 mice were intravenously injected with MSCs, and divided into three groups (PBS, BMMSCs and JMMSCs). Wound healing rate was calculated at different time points (3, 6, 9, 12 day) , and mice were sacrificed on day 12 after treatment and skin samples were harvested for further analysis. **(b)** Scheme illustrating the cutaneous wound procedure and treatment with CM-DIL labeled MSCs. 9 skin defected mice were established, and divided into three groups (PBS, BMMSCs and JMMSCs). Mice were sacrificed on day 7 after treatment and skin samples were harvested for further analysis. **(c)** Scheme illustrating the cutaneous wound procedure and administration of exosomes. 16 skin defected mice were established, and divided into four groups (PBS, BMMSCs, BMMSC-ex and BM/siRab27).

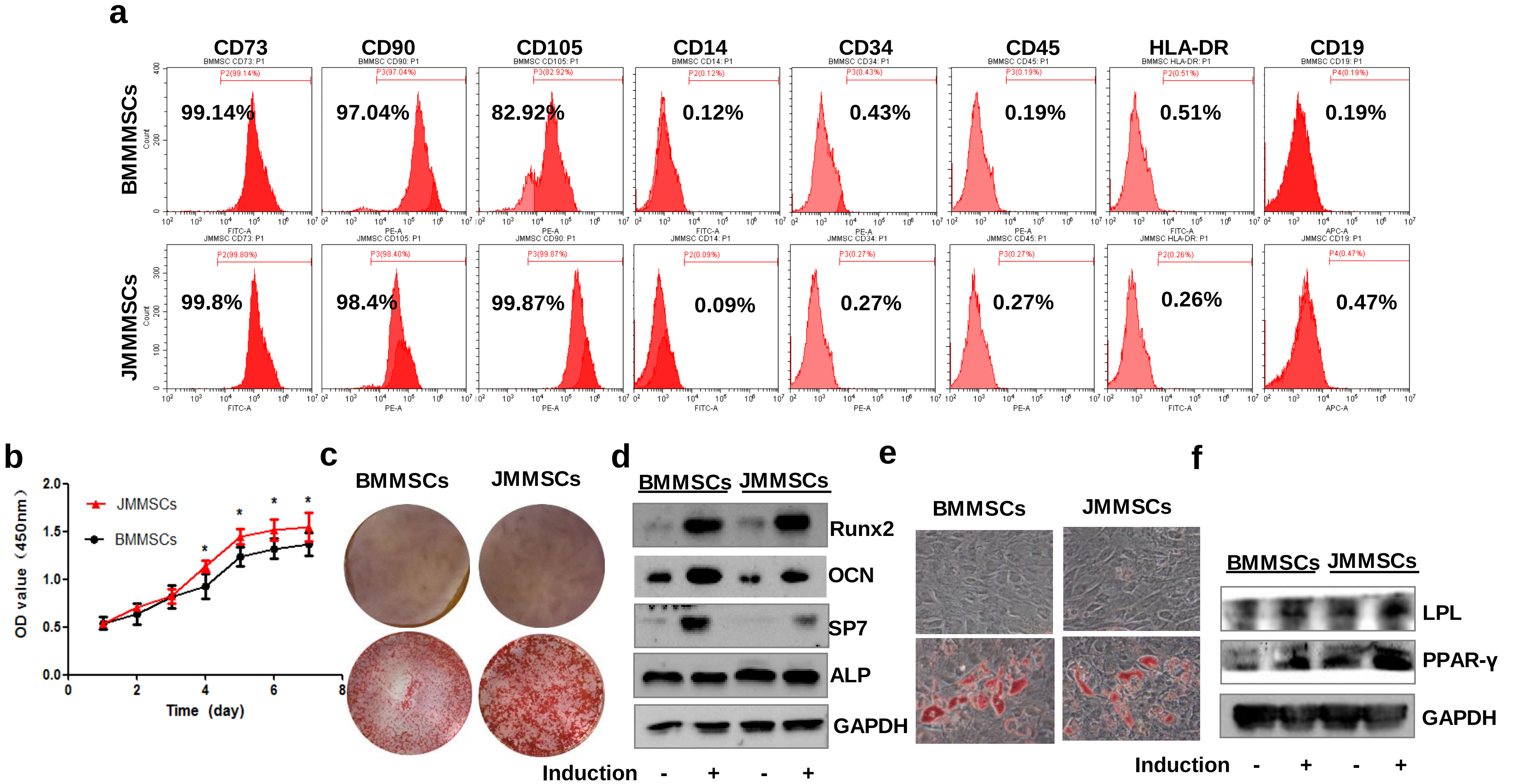

Supplementary Figure S2. Mesenchymal stem cell characteristics of BMMSCs and JMMSCs. **(a)** Flow cytometric analysis of surface markers on BMMSCs and JMMSCs. The results confirmed that these MSCs were positive for the MSC surface markers CD73, CD90, and CD105, but were negative for the hematological markers CD14, CD34, CD45, CD19 and HLA-DR. **(b)** Cell growth curves of BMMSCs and JMMSCs were determined using MTT assay. **(c)** Osteogenic differentiation determined by Alizarin Red staining. The upper panel is non-induced control, and the lower panel is osteogenic induction group. **(d)** Expression of osteogenic differentiation related proteins was determined by western blot (Runx2, OCN, SP7 and ALP). **(e)** Adipogenic differentiation determined by Oil Red O staining. The upper panel is non-induced control, and the lower panel is adipogenic induction group. **(f)** Expression of adipogenic differentiation related proteins was determined by western blot (PPAR-γ and LPL). *$P<0.05$. Error bars are mean ± SD.

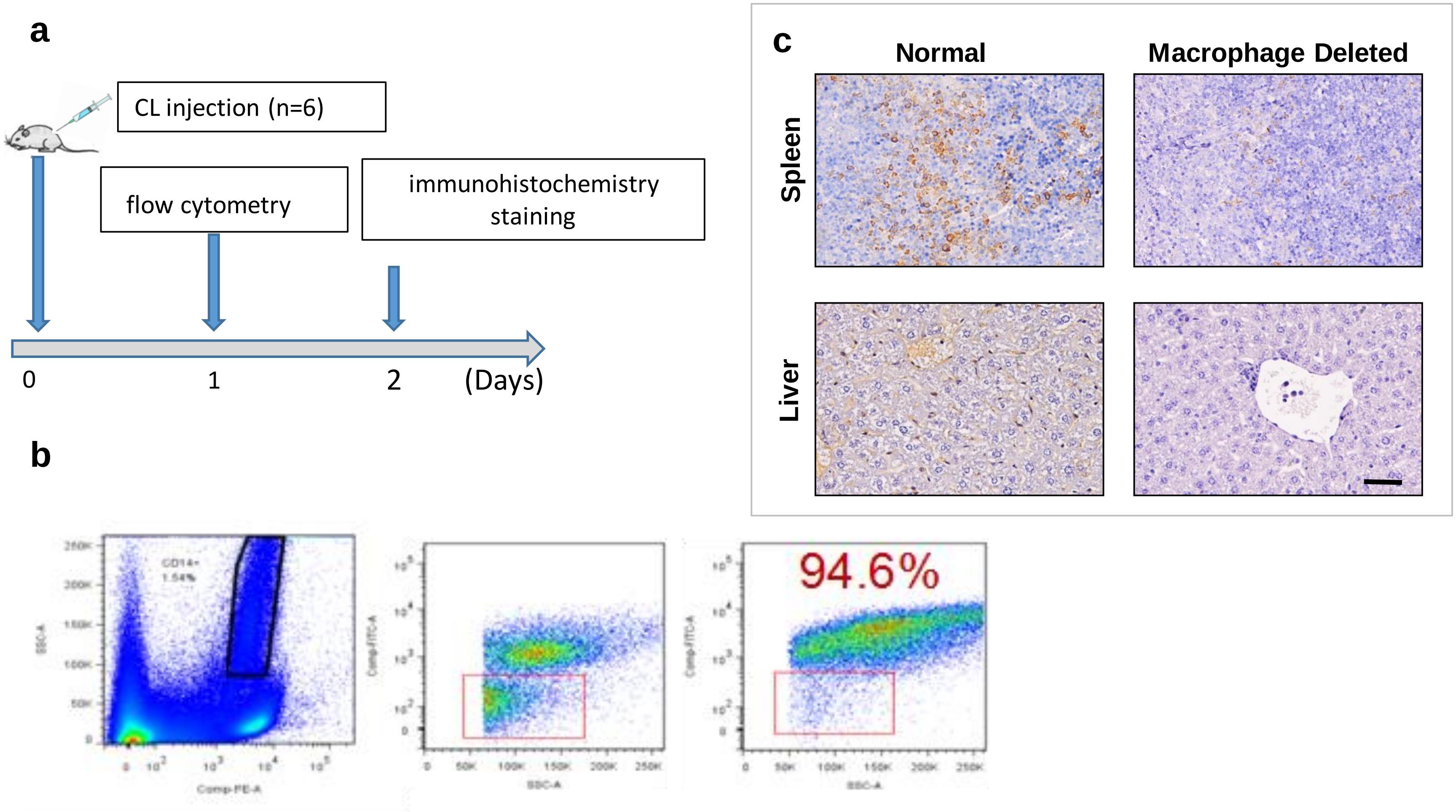

Supplementary Figure S3. Early depletion of macrophages in mice. (a) Scheme illustrating the macrophage deleted procedure. 6 mice were intravenously injected with CL (5 mg/ml, 200 µl per mouse), and 3 mice were sacrificed one day after injection to detect the deletion efficiency by flow cytometry, the remaining mice were sacrificed the next day. **(b)** 94.6% macrophages were depleted assessed by flow cytometry analysis. The cells isolated from blood after 24 h of CL injection, and stained with CD14 (PE-labeled) and CD45 (FITC-labeled) . **(c)** Immunohistochemical staining of CD68 was performed to test the efficiency of macrophages depletion in spleen and liver of mice. Few CD68 positive macrophages was observed in spleen and liver of mice. Scale bar, 100µm.

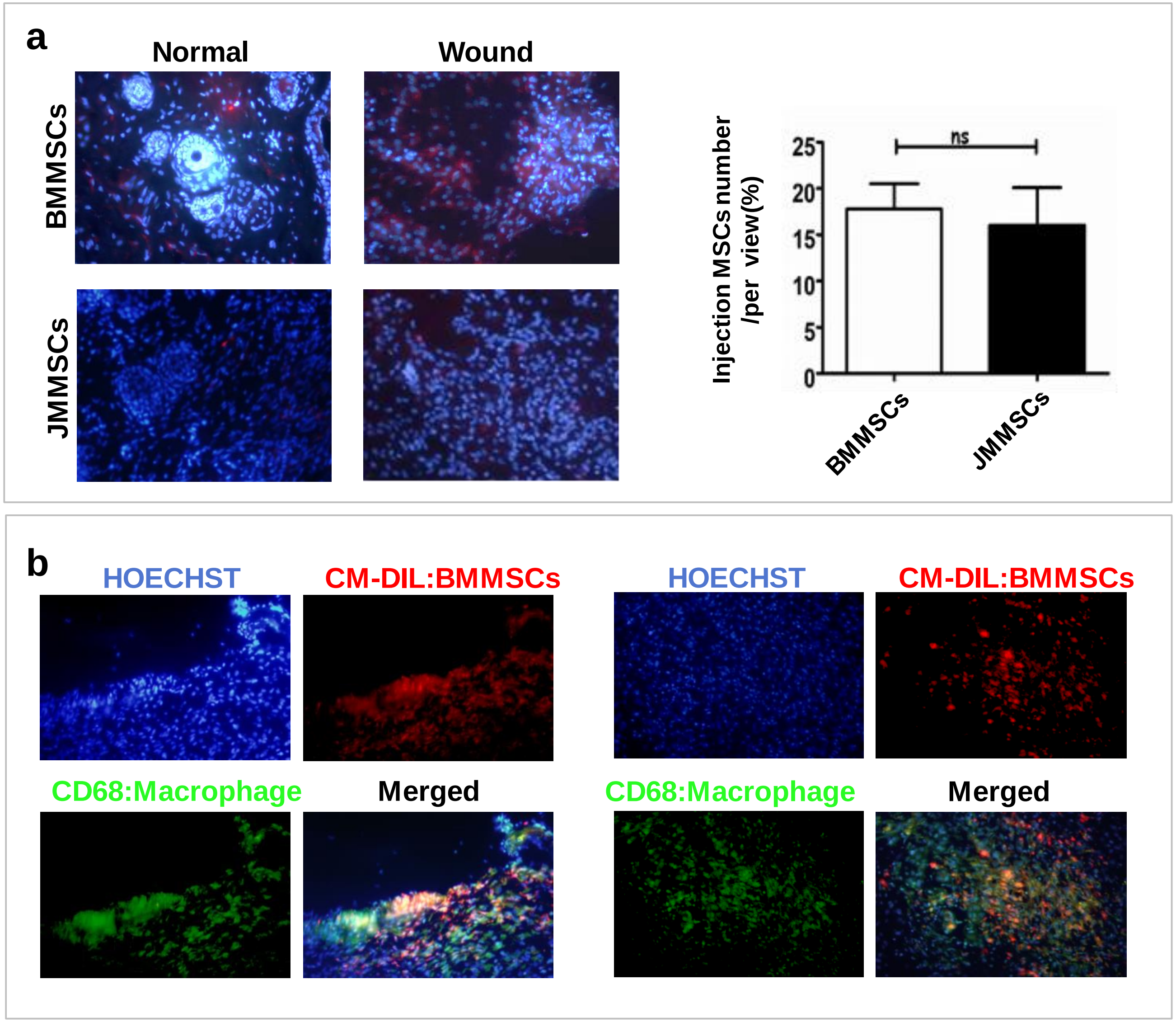

Supplementary Figure S4. MSC home to the wound site and colocalize with cd68+ cells in the wound bed. **(a)** CM-DiL labeled MSCs (red) homing to the wound site (left), the numbers of BMMSCs homing to the wound site had no difference compared to the numbers of JMMSCs (right). **(b)** CM-DiL labeled MSCs (red) and CD68 (geeen) stained macrophages at the wound site.

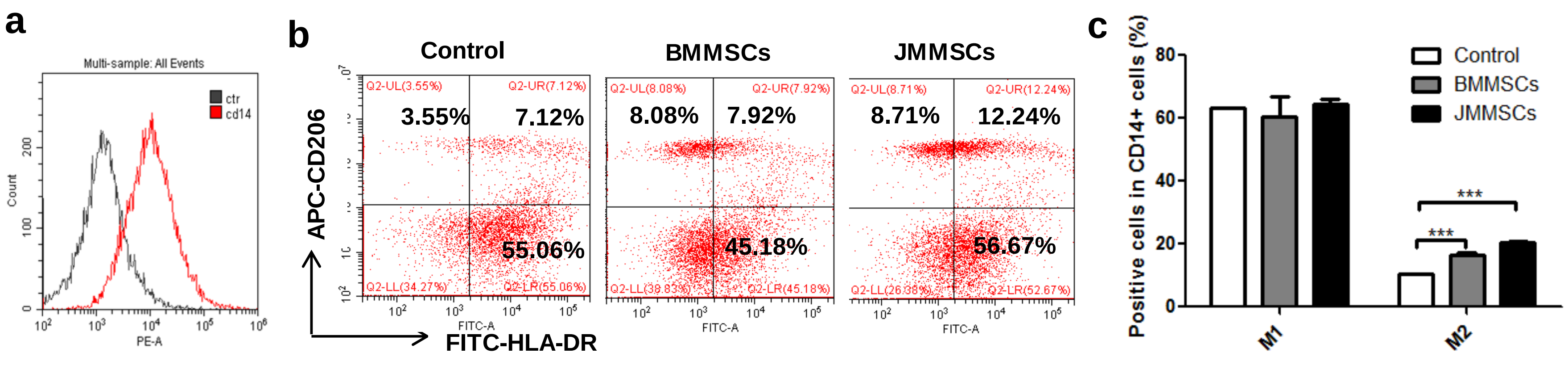

Supplementary Figure S5. M1/M2 after co-cultured with MSCs for 72 hours. **(a)** CD14+ macrophages were sorted with PE-CD14. **(b)** CD206 + and /or HLA-DR + cells were sorted from CD14+ cells by flow cytometry. The results showed that the expression of CD206 in macrophages was increased after co-cultured with BMMSCs or JBMMSCs compared to the control group. But the M1 marker HLA-DR was no significant difference among three groups. **(c)** histogram of M1 and M2 in CD14+ cells (n=3). ***P< 0.001. Error bars are mean ± SD.

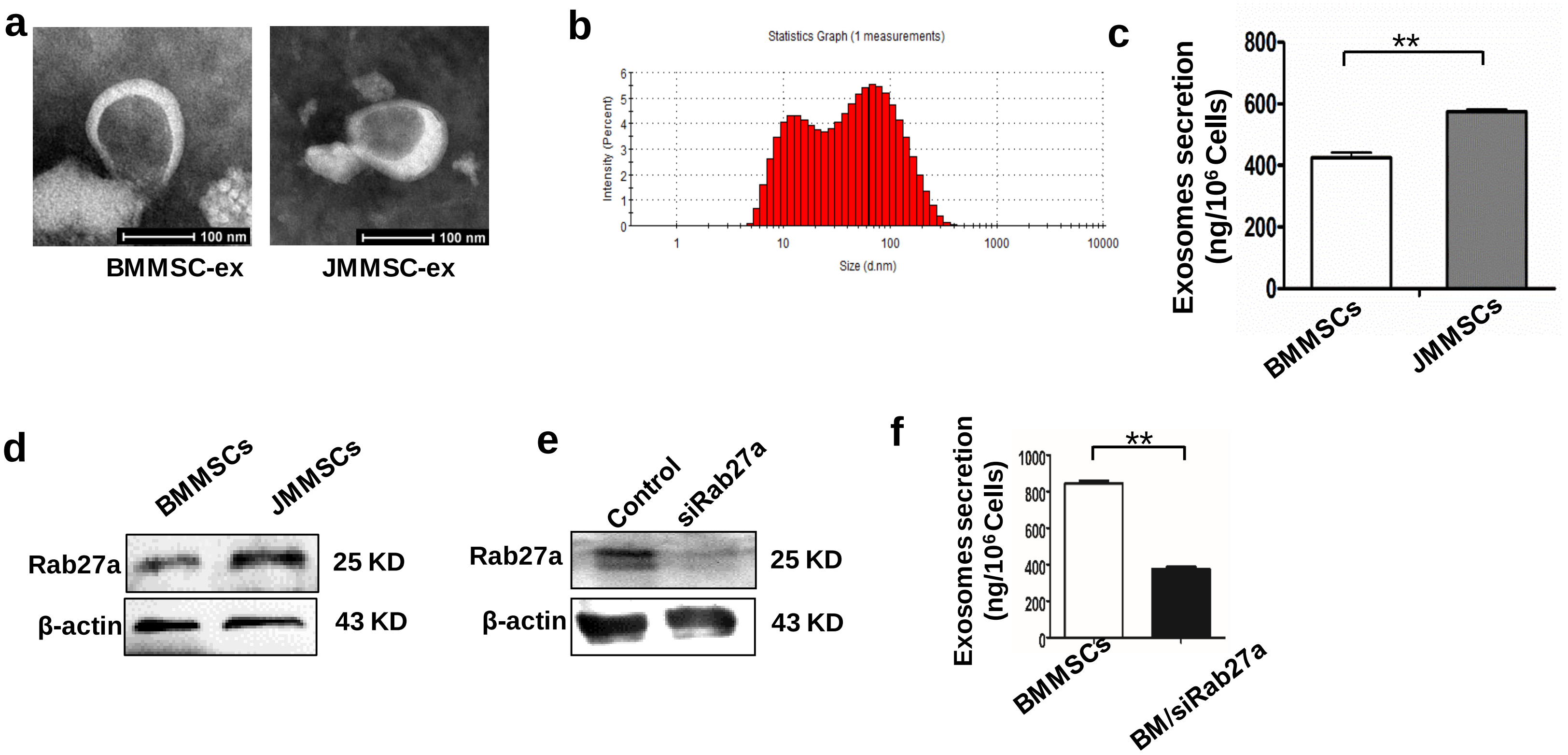

Supplementary Figure S6. Exosomes secreted by MSCs. (a) Transmission electron microscopy showed exosomes from BMMSCs or JBMMSCs. **(b)** Representative size and particle distribution plots of BMMSC-ex. **(c)** Comparison of exosome secretion between BMMSCs and JBMMSCs. **(d)** Western blot analysis of Rab27a in BMMSCs and JMMSCs. **(e)** Western blot analysis was performed to examine the expression of Rab27a after transfecting siRab27a in BMMSCs. **(f)** Secretion of exosomes was measured after knockdown Rab27a in BMMSCs. **P< 0.01. Error bars are mean ± SD.

Table 1
Primers sequences used for polymerase chain reaction amplifications

Gene		sequences(5’-3’)
GAPDH-m	Forward	AGGTCGGTGTGAACGGATTTG
	Reverse	GGGGTCGTTGATGGCAACA
Arg-1-m	Forward	GTGAAGAACCCACGGTCTGT
	Reverse	AGAAAGGACACAGGTTGCCC
TNF-a-m	Forward	AGGCACTCCCCCAAAAGATG
	Reverse	GCTCCTCCACTTGGTGGTTT
GAPDH-h	Forward	GGAGCGAGATCCCTCCAAAAT
	Reverse	GGCTGTTGTCATACTTCTCATGG
IL-10-h	Forward	CGAGATGCCTTCAGCAGAGTG
	Reverse	CCTTGATGTCTGGGTCTTGGTT
TNF-a-h	Forward	CTCCAGGCGGTGCTTGTTC
	Reverse	GGCTACAGGCTTGTCACTCG
MiR-223	Forward	TGTCGTGTCAGTTTGTCAAATAC
	Reverse	GTGCAGGGTCCGAGGTATTC
